# Tadpole serum activity (*Rana catesbeian* a) in caspase-3 as a marker of the role of apoptosis and total cytotoxic T lymphocytes in albino rats’ epithelial cells induced by neoplasia

**DOI:** 10.14202/vetworld.2019.63-67

**Published:** 2019-01-10

**Authors:** M. T. E. Purnama, I. H. Rahmaningtyas, A. R. Pratama, Z. Prastika, A. M. Kartikasari, N. P. D. Cahyo

**Affiliations:** 1 Department of Veterinary Anatomy, Faculty of Veterinary Medicine, Universitas Airlangga, Mulyorejo Campus C Surabaya, East Java 60115, Indonesia; 2 Department of Veterinary Pathology, Faculty of Veterinary Medicine, Universitas Airlangga, Mulyorejo Campus C Surabaya, East Java 60115, Indonesia

**Keywords:** caspase-3, cytotoxic T lymphocyte, *Rana catesbeiana*, serum, tadpoles

## Abstract

**Aim:**

This study was conducted to examine the tadpole’s serum activity (*Rana catesbeiana*) in caspase-3 as a marker of the role of apoptosis and total cytotoxic T lymphocyte (CTL) in albino rats’ epithelial cells induced by neoplasia. Tadpole serumcontains thyroxine hormone that may cause the metamorphosis process and control cell proliferation.

**Materials and Methods:**

Male rats were induced by 7,12-dimethylbenz (α)anthracene (DMBA) 20 mg/rats twice every week over 5 weeks to stimulate skin neoplasia. Tadpole serum injected intracutaneously after neoplasia is known. The negative control group (C−) was not exposed to DMBA and tadpole serum, while the positive control group (C+) was exposed to DMBA. Treatment groups (T1, T2, and T3) were exposed DMBA and tadpole serum 100%, 75%, and 25%/rat/day, respectively. Samples of skin organ were be made preparations immunohistochemistry interacted with caspase-3 and CTL antibody as the marker.

**Results:**

Based on the result, immunohistochemistry from skin neoplasia and given therapy of tadpole serum show that Treatment 1 was the highest caspase-3 and CTL expression. The result of caspase-3 expression in C−, C+, T1, T2, and T3 was 0.00^c^±0.000, 0.70^bc^±0.141, 2.00^a^±0.283, 1.10^b^±0.424, and 1.15^b^±0.495, respectively. The result of CTL expression in C−, C+, T1, T2, and T3 was 0.10^d^±0.200, 1.00^c^±0.230, 2.10^a^±0.529, 1.70^ab^±0.258, and 1.35^bc^±0.443, respectively.

**Conclusion:**

It can be concluded from the study that tadpole serum (*R. catesbeiana*) 100% concentration can increase caspase-3 and total CTL in albino rats’ epithelial cells induced by neoplasia.

## Introduction

Cancer is one of the highest mortality diseases in the world. In 2012, there were 14 million new cases with 8 million mortality rate in the same year [1]. More than 232,000 cases of skin cancer were recorded, and among them, 55,000 cases resulted in the death [2]. Cancer cells are normal cells that undergo a change into malignant due to genetic abnormalities of these cells, so the ability of cell division is out of control. Malignant cells will trigger the growth of proto-oncogene cells so that normal cells will have potentially uncontrolled proliferation [3,4]. Mutation of genes in cancer increases cell proliferation and resistance to apoptosis mechanisms [5]. Some body molecular systems are used to induce apoptosis pathways to inhibit tumor cell growth [6].

Thyroxine hormone leads an important role in different organs and tissues [7]. Thyroxine hormone increases gene expression associated with proliferation, cell differentiation, and development of body shape in tadpoles [8,9]. Thyroxine hormone can induce apoptosis and minimize myogenesis of tadpole in the process of metamorphosis [10]. Apoptosis is programmed cell death. Apoptosis is an important mechanism in embryonic development, organogenesis, and metamorphosis [11]. In the frog metamorphosis, the tail tissue undergoes apoptosis due to an increase in thyroxine hormone [12].

The aim of this study was to examine the potential serum of tadpoles (*Rana catesbeiana*) in caspase-3 as a marker of the role of apoptosis and total cytotoxic T lymphocyte (CTL) in albino rats’ epithelial cells induced by neoplasia. Tadpole serum containing thyroxine hormone is expected to induce the apoptosis process in skin epithelial cells, thus becoming a solution to inhibit the development of proto-oncogene cells.

## Materials and Methods

### Ethical approval

This study was approved by the Ethical Committee with Ethical Clearance No: 686-KE Animal Care and Use Committee, Faculty of Veterinary Medicine, Universitas Airlangga.

### Procedures for 7,12-dimethylbenz (α)anthracene (DMBA) administration

This study was used male rat Sprague Dawley strain of 20 rats weighing 200 g. Adaptation in animals is done for 1 week with a cluster-type enclosure. Healthy condition was determined by their active movement. Dosage induction DMBA (Sigma-Aldrich^®^ with CAS number 57-97-6) as a reagent to induce cancer cells according to research reported as much 20 mg/rats twice every week for 5 weeks [13]. To present skin cancer cells in this study, injected DMBA according to induction dose intracutaneously using a tuberculin syringe. Before induction, DMBA powder was dissolved in corn oil to facilitate the induction process by comparison in 1 ml of corn oil containing 20 mg doses of DMBA.

### Isolation of tadpole serum

Tadpole blood 45-46 days old was collected through intracardial using a tuberculin syringe [14]. To increase the serum volume, the blood was then centrifuged for 15 min at a speed of 1000 rpm. Tadpole serum moved on a tube, covered with aluminum foil, and placed into the refrigerator with the temperature around −15°C-−30°C.

### Procedures for tadpole serum injection

This study was an experimental laboratory using a completely randomized design for 20 rats. They were divided into five treatments in which each treatment was subjected to four replications as follows: Negative control group (C−) was not exposed to DMBA and tadpole serum; positive control group (C+) was exposed to DMBA; treatment groups (T1, T2, and T3) were exposed DMBA and tadpole serum 100%, 75%, and 25%/rat/day, respectively. Tadpole serum was injected intracutaneously in DMBA injected site. Natural killer (NK) cell stimulation was performed by interferon gamma (INF-γ) with thyroxine hormone used a minimal dose of 0.01-0.1 mg/ml [15]. Thyroxine hormone in tadpole serum is 0.94 µg/dl [16]. In this study, we used a therapeutic dose of 0.01 mg/ml with detailed treatment;, Treatment 1 used serum dose of 1.06 ml/rat/day, Treatment 2 used serum dose of 0.71 ml/rat/day and buffer solution 0.35 ml/rat/day, and Treatment 3 used serum dose of 0.27 ml/rat/day and buffer solution 0.79 ml/rat/day.

### Animal surgical procedures

After the dislocation of the ossa cervical atlanto-occipitale, surgery has been done by shaving the hair and skin incision. The skins were separated from the other connective tissue and then stored in formaldehyde 15% for making preparations for immunohistochemistry staining.

### Procedures for immunohistochemistry staining

The skins were stored in formaldehyde 15% for 48 h. Furthermore, alcohol was used as a dehydration agent with concentration of 70%, 80%, and 96%. Xylol was used for clearing process and continued making paraffin block with 60°C of the temperature. The skin tissue that has received paraffin blocks then sliced using a microtome machine and then transferred into a water bath before being placed on a glass object. Immunohistochemistry staining was used primary antibody caspase-3 anti-rat (PE Active Caspase-3 Apoptosis Kit BD Pharmingen^™^ with CAS number 550914) and CTL (ATCC^®^ PCS-800-017^™^) for 1 h in 27°C. The dilution was given 10 µl for caspase-3 and 0.1 ml for CTL test. Caspase-3 was executor primary antibody [17]. Then, the specimen washed in phosphate buffered saline (PBS) with a pH of 7.4 for 3 times every 5 min. The next preparations were added streptavidin-horseradish peroxidase for 60 min in 27°C and washed in PBS with pH 7.4. Then, the specimens were added chromogen 3,3-Diaminobenzidine tetrahydrochloride for 20 min and washed with aquadest for 5 min.

### Observations of skin scoring with immunohistochemistry staining

The observations were focused on the caspase-3 and CTL expression of skin epithelial cell. The intensity of the visual field brownish 0% with a score of 0 means normal, intensity of 0-25% of the visual field with a score of 1 means low, intensity 25-50% of the visual field with a score of 2 means medium, and the intensity of the visual field >50% with a score of 3 means high [18]. Scoring was done as many as 10 of the visual field in each replication with a magnification of 1000 times, and the observations were made with a microscope Nikon^®^ E-200 LED Trinocular Microscope.

### Statistical analysis

The mean score of the observations was tested by Kolmogorov-Smirnov for the normality test. If the data normal distribution, then continued with one-way ANOVA test and if a significant difference (p<0.05) was found, the analysis was followed with Duncan’s test. All the processes of analyses were used SPSS v21 software (IBM, USA).

## Results

**Figure-1:**
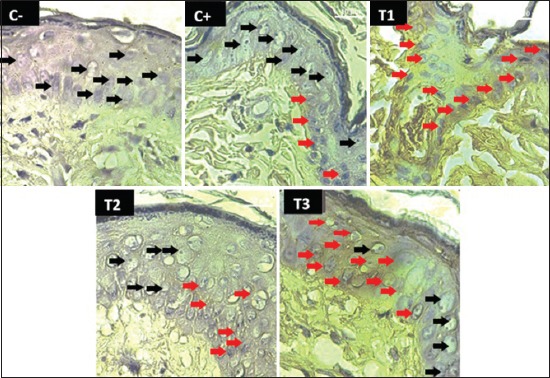
Caspase-3 expression in the treatments of C−, C+, T1, T2, and T3. (→) The black arrow shows the normal skin epithelial cell, (→) the red arrow shows the caspase-3 expression of skin epithelial cell.

**Figure-2:**
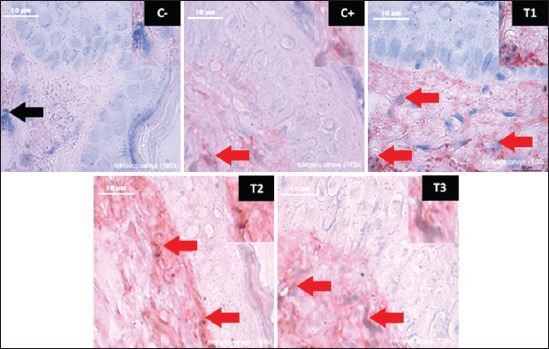
Cytotoxic T lymphocyte (CTL) expression in the treatments of C−, C+, T1, T2, and T3. (→) The black arrow shows the normal T lymphocyte, (→) the red arrow shows CTL expression on skin epithelial cell.

The results of the observation showed that the brownish color was qualitative with the caspase-3 antibody primer and CTL expression. Table-1 shows the mean and SD with a significant difference between treatments (p<0.05). Treatment 1 was an effective given 100% tadpole serum as the highest score caspase-3 and CTL expression followed by Treatment 2 of 75% tadpole serum and Treatment 3 of 25% tadpole serum. Caspase-3 (Figure-1) and CTL (Figure-2) show the different color intensity of skin epithelial cell between each treatment. This means that tadpole serum 100% can induce apoptosis process on skin epithelial cell albino rats induced by neoplasia.

**Table-1:** Mean and SD caspase-3 and CTL expression.

Treatments	Mean±SD	

	Caspase-3	CTL
C−	0.00^c^±0.000	0.10^d^±0.200
C+	0.70^bc^±0.141	1.00^c^±0.230
T1	2.00^a^±0.283	2.10^a^±0.529
T2	1.10^b^±0.424	1.70^ab^±0.258
T3	1.15^b^±0.495	1.35^bc^±0.443

Different superscripts in the same column indicate significant differences among treatments (p<0.05), CTL=Cytotoxic T lymphocyte, SD=Standard deviation

## Discussion

Based on Table-1, Treatment 1 shown 2.00^a^±0.283 as the highest value of apoptosis occurrence because this treatment was given a 100% dose of tadpole serum. Apoptosis occurrence is indicated by an assessment of the caspase-3. If compared with positive control group (C+) only induced by DMBA without treatment tadpole serum, the apoptosis score was only 0.70^bc^±0.141 for caspase-3. Treatments 2 and 3 have a score of 1.10^b^±0.424 and 1.15^b^±0.495 smaller than Treatment 1.

Based on Table-1, results of the mean Treatment 1 indicate the mean of CTL 2.10^a^±0.529, in which Treatment 2 is 1.70^ab^±0.258, and Treatment 3 is 1.35^bc^±0.443. The significant difference occurred between Treatment 1 and Treatment 3 but no significant between Treatment 1 with Treatment 2 and Treatment 2 with Treatment 3.

Tadpole on stage 21-23 days old contains thyroxine hormone in blood serum. The concentration of thyroxine hormone contains 0.94 µg/dl [16]. The thyroxine hormone was measured by radioimmunoassay double antibodies in blood serum and pericardial fluid bullfrog tadpoles. Tadpole on stage 5-18 days old also has the thyroxine hormone concentrations <0.20 mg/dl and began to be detected in its initial of metamorphosis stage 19-20 days old [19].

The thyroxine hormone leads a role in tadpole apoptosis, so tail regression occurs. [19]. This is also supported by the role of thyroxine hormone in the metamorphosis of tadpole [3]. Thyroxine hormone has a function in apoptosis of the frog, so the regression occurs in the tail [20-22]. This invention applied in this research so that serum which is contained with thyroxine hormone can inhibit proto-oncogene cell proliferation. Thyroxine hormone can increase humoral and cellular immune response. Humoral immune response increases the number of NK cells while the cellular immune response increases the number of CTL [23]. Both cells worked specifically to inhibit proto-oncogene cell proliferation and killed cancer cells so that the apoptosis process happened [24-27].

Apoptosis of skin cancer cells is shown in caspase-3 (Figure-1) and CTL (Figure-2) with brownish color on skin epithelial cells. CTLs are a type of white blood cell that can prevent intracellular pathogens and become a marker of the process of the phagocytosis of cancer cells. Apoptosis happened because CTL and NK cells kill cancer cells [28,29]. Apoptosis can be through extrinsic pathways in death receptor and intrinsic pathways or mitochondrial pathways. Through extrinsic pathways, there is a relationship between ligand and death receptor so that can activate sequential caspase-8 as the initiator of the apoptosis process [30-33]. Tumor necrosis factor (TNF) which consists of cytoplasmic domains is called the death domain. Death domains will transmit apoptotic signals through TNF receptor Type-1 and connect to a Fas protein (CD95) that will bind to Fas ligand. Cytoplasmic Death Domain forms the binding site as a Fas-associated death domain (FADD) protein adapter. The FADD protein will bind to pro-caspase 8 to activate caspase-8 [30,34]. Caspase-8 will initiate the release of caspase-3 which acts as the executor of the apoptosis process [17].

Increasing the number of CTL occurs due to the influence of tadpole serum (*R. catesbeiana*) containing the hormone thyroxine. Dendritic cells can help the thyroid hormone as a cancer vaccine to stimulate CTL as immunotherapy [7]. Other studies using L-thyroxine (T_4_) and 3,3,5-triido-L-thyronine (T_3_) could stimulate the proliferation of T lymphocytes [23]. Thyroxine hormone that used low doses could increase the stimulatory effect of IFN-γ [15]. Giving triiodothyronine was able to stimulate an antitumor CTL response [12].

The thyroid gland regulates the immune system indirectly by released hormones and cytokines. The cytokines include IFN-γ (15). 3,3,5-Triido-L-thyronine (T_3_) and L-thyroxine (T_4_) could modulate specific immune response, issued cell-mediated immunity, NK cell activity, as a result of IFN antiviral response and the proliferation of T lymphocytes [34].

## Conclusion

Tadpole serum (*R. catesbeiana*) 100% concentration can increase caspase-3 and total CTL in albino rats’ epithelial cells induced by neoplasia. Tadpole serum can be used as a cancer therapy with further research.

## Authors’ Contributions

MTEP and IHR supervised the experiment. ARP and NPDC helped in immunohistochemistry method. IHR, AMK, and ZP conducted the study. MTEP helped in the statistical analysis of the data. MTEP, IHR, and ARP helped in the preparation of tables and figure, revised, and submitted the manuscript. All authors read and approved the final manuscript.
